# Quantitative Characterization of the Influence of the Nanoscale Morphology of Nanostructured Surfaces on Bacterial Adhesion and Biofilm Formation

**DOI:** 10.1371/journal.pone.0025029

**Published:** 2011-09-26

**Authors:** Ajay Vikram Singh, Varun Vyas, Rajendra Patil, Vimal Sharma, Pasquale Emanuele Scopelliti, Gero Bongiorno, Alessandro Podestà, Cristina Lenardi, Wasudev Namdev Gade, Paolo Milani

**Affiliations:** 1 European School of Molecular Medicine (SEMM), IFOM-IEO, Milan, Italy; 2 Interdisciplinary Centre for Nanostructured Materials and Interfaces (CIMAINA), Department of Physics, Università degli Studi di Milano, Milano, Italy; 3 Department of Biotechnology, University of Pune, Ganesh Khind, Pune, India; 4 Fondazione Filarete, Milano, Italy; RMIT University, Australia

## Abstract

Bacterial infection of implants and prosthetic devices is one of the most common causes of implant failure. The nanostructured surface of biocompatible materials strongly influences the adhesion and proliferation of mammalian cells on solid substrates. The observation of this phenomenon has led to an increased effort to develop new strategies to prevent bacterial adhesion and biofilm formation, primarily through nanoengineering the topology of the materials used in implantable devices. While several studies have demonstrated the influence of nanoscale surface morphology on prokaryotic cell attachment, none have provided a quantitative understanding of this phenomenon. Using supersonic cluster beam deposition, we produced nanostructured titania thin films with controlled and reproducible nanoscale morphology respectively. We characterized the surface morphology; composition and wettability by means of atomic force microscopy, X-ray photoemission spectroscopy and contact angle measurements. We studied how protein adsorption is influenced by the physico-chemical surface parameters. Lastly, we characterized *Escherichia coli* and *Staphylococcus aureus* adhesion on nanostructured titania surfaces. Our results show that the increase in surface pore aspect ratio and volume, related to the increase of surface roughness, improves protein adsorption, which in turn downplays bacterial adhesion and biofilm formation. As roughness increases up to about 20 nm, bacterial adhesion and biofilm formation are enhanced; the further increase of roughness causes a significant decrease of bacterial adhesion and inhibits biofilm formation. We interpret the observed trend in bacterial adhesion as the combined effect of passivation and flattening effects induced by morphology-dependent protein adsorption. Our findings demonstrate that bacterial adhesion and biofilm formation on nanostructured titanium oxide surfaces are significantly influenced by nanoscale morphological features. The quantitative information, provided by this study about the relation between surface nanoscale morphology and bacterial adhesion points towards the rational design of implant surfaces that control or inhibit bacterial adhesion and biofilm formation.

## Introduction

Biomedical implants and smart prosthetics increasingly incorporate engineered surfaces at the nanoscale in order to modulate and control the interaction between biomaterials and biological systems [1]–[3]. Among various inorganic and organic materials used for implants and prosthetics, titanium oxide is the most widely used for orthopedic and dental implants, because of its excellent biocompatibility, mechanical strength and chemical stability [4]–[7]. A large number of studies qualitatively demonstrate that nanostructures on titanium oxide surface can enhance cell adhesion and proliferation [8]–[10]. Yet no quantitative understanding of the role of nanoscale morphology on cell behavior exists. It is believed that protein adsorption could be the key factor that determines the different behavior of cells on nanostructured surfaces [11]–[13]. In fact, when a biomaterial surface comes into contact with biological fluids, such as blood or serum, it is immediately coated by the proteins present in the media. This protein layer strongly influences cell adhesion and proliferation on implants. Protein-surface interaction is determined by the complex interplay between morphological and chemical features. These include surface charge, hydrophobicity, roughness and chemical composition [14]–[15]. The quantitative study of protein adsorption on nanostructured surfaces, in terms of separating the role of parameters, such as surface chemistry and surface nanotopography, has been a recent development [15].

The study of bacterial adhesion and proliferation on surfaces is as critical as the study of eukaryotic cell attachment for evaluating materials performance for biomedical applications. Despite its significance, very few studies have been devoted to understanding how titanium oxide nanoscale morphology affects surface-bacteria interactions *in vitro*. In particular, attempts are made to comprehend the existing correlation among surface morphology, amount of adsorbed proteins and bacteria adhesion [16]. This understanding could be of fundamental importance for the rational design of implant surfaces able to promote mammalian cell function and inhibit bacterial colonization and biofilm formation simultaneously [8].

Previous research findings illustrate that the presence of nanostructures on surfaces generally promotes bacterial adhesion and biofilm formation. Truong et al. have shown that the adhesion of bacterial cells on titanium surfaces is promoted by the presence of nanoscale topographical features [17]. Whitehead et al. have studied bacterial colonization on nanostructured titanium surfaces, and demonstrated improved colonization efficiency when surface roughness increases [18]. Similar trends have been reported by Bakker et al. for polymer surfaces with nanometre scale roughness [19]. Puckert et al. as well have recently studied the correlation between bacterial adhesion and the spatial organization of nano-features of different shape and sizes on TiO_2_ surfaces [20].

These studies give a qualitative view of the interaction between nanostructured surfaces and bacteria, however, a quantitative understanding is still lacking. In fact, these studies have been carried out on surfaces modified by chemical etching or mechanical roughening, with little quantitative control on the nanoscale features and on surface chemistry. Moreover, no attention has been dedicated to the study of protein adsorption on the nanostructured samples, nor to the possible influence of the adsorbed proteins on bacteria adhesion and biofilm formation.

In this article we present a quantitative experimental strategy to study the interaction between bacteria and nanostructured surfaces, and to correlate surface morphological and chemical properties with the amount of adsorbed proteins as well as with bacterial adhesion. Firstly, we used supersonic cluster beam deposition (SCBD) in order to produce nanostructured titania samples (ns-TiO_2_) with controlled nanoscale morphology of varying surface root-mean-squared (rms) roughness from 16 nm to 32 nm. Secondly, we quantitatively characterized surface morphology by using atomic force microscopy (AFM) and surface chemical characteristics by performing X-ray photoelectron spectroscopy (XPS), contact angle and surface energy measurements. Then, we measured the amount of adsorbed proteins on the different nanostructured samples, in order to study how surface morphology and surface chemistry affect the formation of the adsorbed protein layer. Lastly, we quantitatively characterized *E.coli* and *S.aureus* adhesion on nanostructured surfaces with different roughness using confocal microscopy, obtaining the number of adhered cells and the bacterial biofilm parameters as a function of nanometre-scale roughness.

## Results

### Production of nanostructured titania films

We produced four different types of ns-TiOx surfaces (SMP1–SMP4) with increasing film thickness (from 50 nm to 300 nm) using a SCBD apparatus equipped with pulsed microplasma cluster source (PMCS) [21]–[23]. The SCBD deposited samples returned four varying morphologies characterized by a root-mean-square surface roughness (Rq), ranging from 16 nm to 32 nm (Table 1). These surfaces are ideal tools for studying the interaction between biological systems and nanostructured surfaces. In fact, the ballistic deposition of TiO_2_ nanoparticles onto a solid surface (bottom-up approach) produces nanostructured films where the surface morphology develops independently from surface chemistry. In particular, film roughness and other morphological parameters can be varied in a broad range by simply changing the thickness of the deposited films, without changing their surface chemistry [24]–[26].

**Table 1:** Surface morphological parameters of ns-TiO_2_ films.

Sample	Thickness (nm)	Rq (nm)	A_spec_	R_sk_	R_ku_
SMP1	50 nm	16.2±0.8	1.63±0.07	1.91±0.2	8.71±3.3
SMP2	100 nm	21.7±1.1	1.71±0.08	2.45±0.2	17.8±1.9
SMP3	200 nm	25.5±1.6	1.82±0.03	4.61±1.1	32.8±8.0
SMP4	300 nm	32.2±0.5	1.91±0.07	6.28±1.8	46±19.11
C (glass)	170 µm	5.12±0.4	1.04±0.07	0.334±0.1	3.72±1.5

### Characterization of surface wettability and composition of ns-TiO_2_ films

Surface chemical composition, surface energy (SE) and surface water contact angle (WCA) are important surface parameters that may have a crucial influence on the interaction of biomaterial surfaces with proteins and cells. In this study, these properties were characterized by means of X-ray Photoemission Spectroscopy (XPS) and contact angle measurements.

XPS was used to study the electronic structure of cluster assembled ns-TiO_2_ films for different surface morphologies. Preliminary XPS spectra, that covers the whole accessible kinetic energy range (data not shown), exhibit intense photoelectron signals of titanium and oxygen and a small contribution of carbon, the presence of which results from air contaminants, such as carbon oxides and hydrocarbons. No in-vacuum sample preparation treatments were conducted to remove surface contaminants, in order to produce and investigate similar surface supports that are used in cell culture experiments.

For each sample, high-resolution spectra were also obtained. No significant differences were observed for the various samples. Figure S1 shows a typical O 1*s* photoemission signal, which is composed by a main peak at 530.3 eV (FWHM = 1.3 eV) related to oxygen bonded to titanium and a small broad shoulder at higher binding energies. This is primarily due to the usual oxygen sources contaminants, such as carbon oxides and physisorbed water. The presence of contaminants prevents the possibility the further study of the O 1*s* line shape, in terms of the presence of different kinds of chemical point defects (O vacancy vs hydroxyls groups).

In Figure S1 a typical high-resolution photoelectron signal of Ti 2*p* spin-orbit (1/2 and 3/2) doublet is also reported. The peak positions in binding energy fall at 458.9 (FWHM = 1.2 eV) and 464.6 eV (FWHM = 2.0 eV) respectively. We did not observe a shift of shoulders towards lower binding energies, attesting to a negligible contribution of TiO_2−x_ oxides or Ti–OH surface groups, and therefore, there exists no direct evidence of Ti^3+^ point defects. These observations, together with the quantitative evaluation of the content of titanium and oxygen bonded to titanium, suggest that the film surface stoichiometry is strictly close to TiO_2_ and confirm the almost complete oxidation of the samples also for moderate annealing.

SE and contact angle of water, glycerol and diidomethane (control solvents) for the nanostructured surfaces used in this study are summarized in Table 2. Water droplets sitting on top of the four ns-TiO_2_ films and corresponding CAs are shown in Figure S2, while WCA and the surface morphology of the reference glass substrate are shown in Figure S3. Table 1 shows the evolution of roughness, WCA and SE for samples of increasing thickness (SMP1–SMP4). We observed an increase of SE and a concurrent decrease in WCA when the roughness was increased. This is not surprising; according to Wenzel law, roughness enhances the wetting character of intrinsically hydrophilic surfaces [27]. Similar variations were also observed for the two control solvents (Glycerol and Diidomethane). Although variations of WCA are significant (up to 35% from the thinnest to the thickest film), they do not represent a change in the wetting character of the film surfaces (no hydrophilic-hydrophobic transitions are induced by roughness).

**Table 2:** Contact angle and Surface energy of ns-TiO_2_ films.

Sample	WCA (°)	GCA (°)	DCA (°)	Surface energy (mj/m^2^)
SMP1	57.8±1.5	69.5±2.1	73.0±3.3	37.7
SMP2	45.9±1.2	50.2±3.3	56.4±3.7	43.1
SMP3	42.4±1.08	42.4±1.08	45.3±2.9	49.3
SMP4	38.5±1.02	44.8±1.5	51.6±3.5	58.6
C (glass)	20.1±1.6	24.4±2.4	29.9±2.2	17.2

WCA/GC/ DCA: Water/Glycerol/Diidomethane Contact Angle.

### Quantitative Morphological Characterization of ns-TiO_2_ films

**Figure 1:**
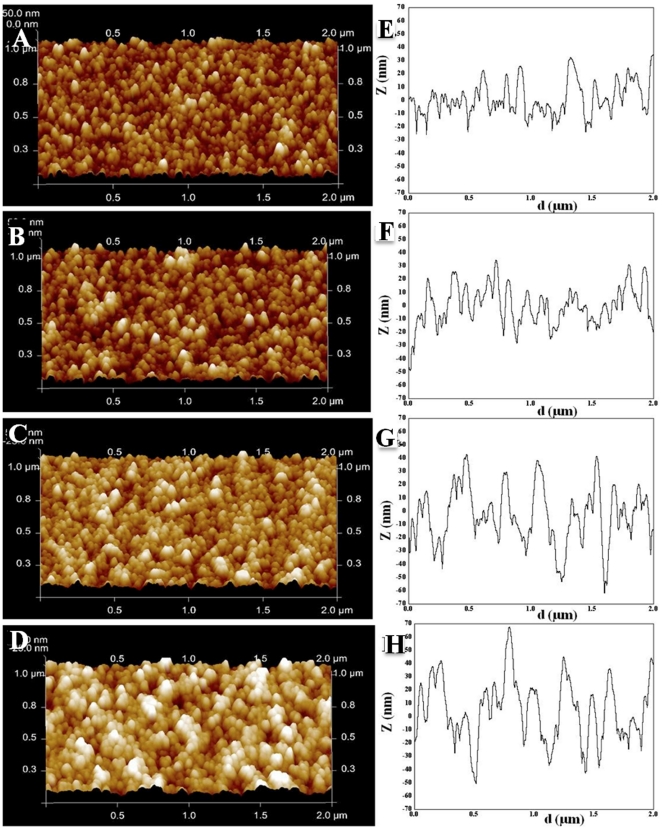
AFM characterization of surface topography of different ns-TiO_2_ films. A–D) Representative height maps in three-dimensional view of ns-TiO_2_ films with increasing thickness (50, 100, 200, and 300 nm); E–H) Representative surface profiles exhibiting variations in Rq, Aspec, correlation length, skewness and kurtosis, as well as in pore width and depth distributions, as discussed in the main text. All images correspond to 2 µm×1 µm scan area.

Representative AFM topographic maps of the ns-TiO_2_ samples are shown in Figure 1A–D, with corresponding representative surface profiles shown in Figure 1E–H. Surface morphology is characterized by a highly porous and granular structure typical of cluster-assembled films [26], [28], with grains diameter ranging from few nm up to 50 nm. The smallest among the grains are primeval clusters, while the others are aggregates of clusters partially coalesced and whose finer structure cannot be resolved by AFM. Both 3-dimensional AFM maps and surface profiles clearly show that during film growth, the evolving surface front not only spreads vertically, which contributes increasing surface roughness, but also develops laterally. The average lateral size of morphological features (correlation length) increases as well as rms roughness when the film thickness increases. The ratio of roughness and correlation length determines the average slope and volume of surface pores. Noticeably, such a complex, nanostructured surface morphology closely resembles that of many biological systems, such as the extra cellular matrix (ECM), and the cell membrane [10], [29]. These peculiar morphological properties have been demonstrated to have a strong impact on protein adsorption and cell response [15], [30].

From AFM topographic maps, several morphological parameters for the four samples were calculated (rms roughness R_q_, specific area A_spec_, skewness R_sk_ and Kurtosis R_ku_), and reported in Table 1. As expected, R_q_ and A_spec_ increase when film thickness increases (the increase is almost linear with film thickness). R_sk_ and R_ku_ values are also increasing functions of the film thickness (t = 0.025, p≤0.05). In particular, the positive increasing values of skewness highlight an asymmetric long-tailed height distribution characterized by protruding asperities and shallow valleys. The R_ku_ values larger than 3 shows progressively peaked surfaces that are decorated by protrusions with high aspect ratio.

The gain in available surface area in the thickest film with respect to a smooth substrate is noticeable (∼100%). As discussed later, the increase in protein adsorption is not directly correlated to the increase of surface roughness (specific area) [15]. This suggests that surface morphology drives this process in a way that is not simply related to the increase of surface roughness (or specific area), but to a more complex interplay of morphological parameters. In particular to the average surface slope and pore volume, which depend not only on the vertical extension of the interface (R_q_), but also on the lateral extension of surface features (correlation length).

Surface profiles of ns-TiO_2_ samples are characterized by nanometric pores of diverse depths and widths (Figure 1). It is demonstrated that the arrangement and the dimensions of surface nanometric pores are fundamental morphological parameters that significantly influence nanostructured surfaces interaction with proteins and cells [15], [31]–[32]. Ns-TiO_2_ pore depth distribution depends on film thickness. In fact, previous investigations [15] showed that films with higher thickness are characterized by a broader pore depth distribution with a greater population in the higher pore depth range (this can be visually discerned in Figure 1). Pores arrangement on the surface (random arrangement) and pore width distribution are instead almost independent from film thickness, and they remains almost constant when film thickness (roughness) is increased. Therefore, the increase of film roughness is correlated with the increase of pore aspect ratio. According to our analysis, aspect ratio of nanopores turns out to be the key morphological parameter driving the interaction of proteins with corrugated surfaces.

### Protein Adsorption

**Figure 2:**
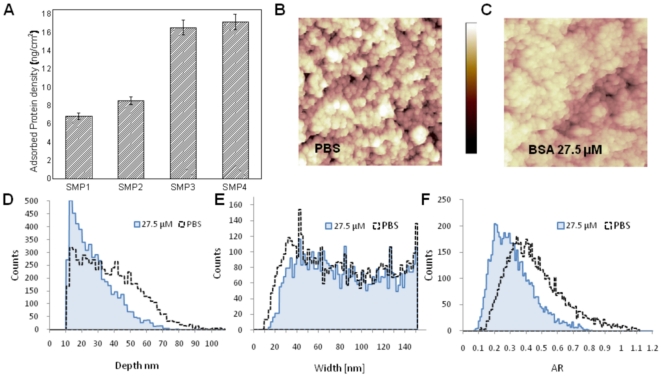
Quantification of protein adsorption on nanostructured thin films. A) FBS adsorption profile on ns-TiO_2_ films. Values shown are mean ± SEM; n = 3. B,C) Representative AFM topographies of the surface of a 300 nm thick ns-TiO_2_ film before and after adsorption of BSA protein. For sake of better comparison, the sample without protein has been incubated for the same time with the protein buffer alone (PBS). D–F) Histogram of depth, width, and aspect ratio of surface pores of ns-TiO_2_ film, calculated as described in details in Ref. [15].

Recently, the group at CIMAINA has widely investigated the effect of nanoscale morphology of ns-TiO_2_ on protein adsorption [15], [33]–[34]. We developed a novel technique to study quantitatively protein-surface interaction using high throughput approach and we elucidated that the increase of nanoscale surface roughness causes a significant increase of the amount of adsorbed proteins [15]. Results presented in this study confirm this trend. The amount of adsorbed FBS on ns-TiO_2_ samples after 30 minutes incubation is shown in Figure 2A. When surface roughness increases, there is a statistically significant increase of protein adsorption on nanostructured samples SMP3–SMP4 compared with SMP1–SMP2. However, while specific area increases by a factor of ∼1.2 from the thinnest to the thickest film, the amount of adsorbed protein increases more rapidly, by a factor of ∼2.5. In previous studies, we demonstrated that the increase of the amount of adsorbed proteins is correlated with the increase of surface pores aspect ratio [15]. In particular, we have shown that proteins accumulate inside nanometric pores that have aspect ratios higher than a certain threshold value (that depends on the protein). This effect is shown in Figure 2B–F, reporting the results of a study of the adsorption of Bovine Serum Albumin (BSA, the most abundant protein in FBS) on ns-TiO_2_ films. BSA adsorption has been characterized by investigating by AFM the morphology of ns-TiO_2_ films before and after BSA incubation. After adsorption of BSA at 27.5 µM sample roughness is significantly lower than before adsorption (Figure 2B–C). The distributions of pore width are very similar before and after adsorption (Figure 2E), while the depth distribution after protein adsorption is different (Figure 2D). It turns out that after adsorption; the depth of deep pores is remarkably reduced, determining a lower aspect ratio on average (Figure 2F). In addition, the depth spectrum is substantially compressed to the lowest depth region, showing that part of the surface pores are filled or partially filled by proteins. These results confirm that proteins aggregate inside surface nanometric pores. For BSA aggregation tends to occur preferentially in pores with aspect ratio higher than 0.4, given that 75% of those pores were partially filled [15]. This indicates that aggregation happen more frequently inside pores with higher aspect ratio.

### Bacterial attachment on nanostructured samples

**Figure 3:**
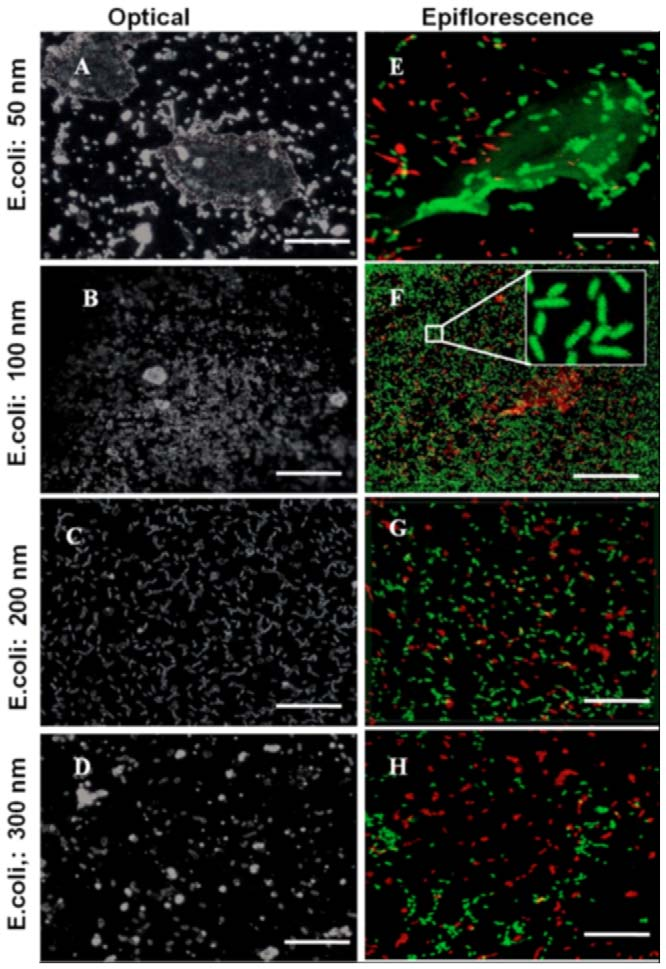
Optical and epifluorescence microscopic images of live/dead *E.coli* species. A–D: Optical microscopy images. E–H: CLSM epifluorescence images of cells stained with BacLight Live/Dead staining kit. Live cells are stained with green and dead cells with red. Inset magnified eightfold, shows magnified view of the biofilm (All images scale bar: 25 µm; Image F: 50 µm).

**Figure 4:**
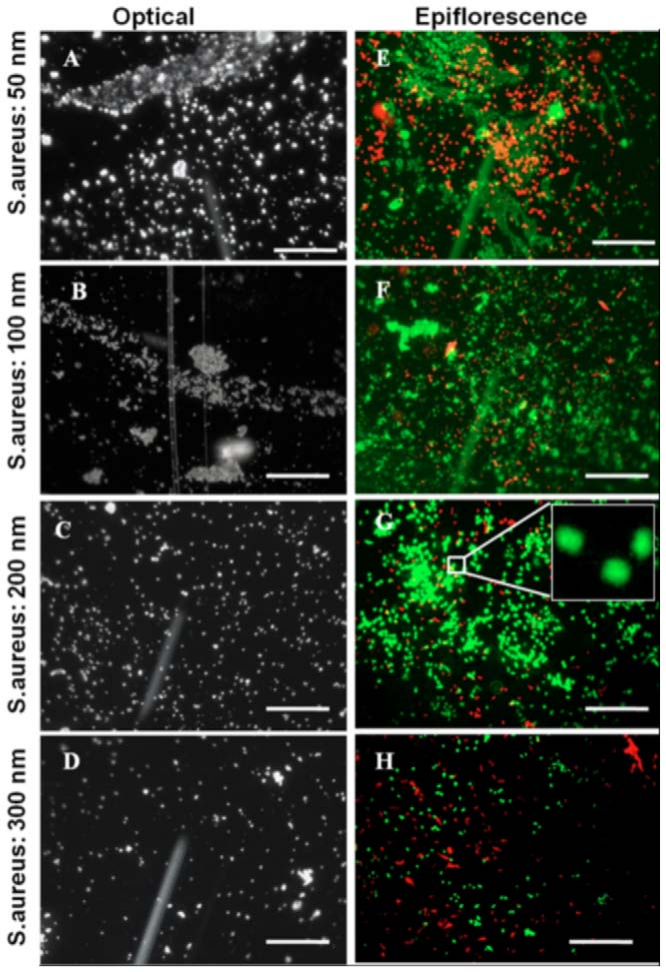
Optical and epifluorescence microscopic images of live/dead *S.aureus* species. A–D. Optical microscopic images. E–H. CLSM epifluorescence images of cells stained with BacLight Live/Dead staining kit. Inset magnified tenfold, shows magnified view of a segment of biofilm (scale bar :25 µm).

We characterized *E.coli* and *S.aureus* adhesion on ns-TiO_2_ surfaces by confocal laser scanning microscopy (CLSM). Representative biofilm structures after 48 hrs for the two species are presented in Figure 3 (*E.coli*) and Figure 4 (*S.aureus*).

Figure 3A–D shows optical microscopy images of *E.coli* cells deposited on nanostructured titania surfaces, where it is possible to notice the formation of a compact biofilm on sample SMP1 and SMP2 (less rough samples). Biofilm is instead absent on samples SMP3 and SMP4 (rougher samples), where there is also a relatively small number of adhered cells. Figure 3E–H shows epifluorescence microscopic images of live (green) and dead (red) *E.coli* cells attached on ns-TiO_2_ films. In these images, it is possible to see that on the less rough samples, live *E.coli* cells (green stained) are embedded in a thin film of extracellular polymeric substance (EPS), which is also immunostained in light green, while on samples SMP3 and SMP4 the EPS film is absent. Movie S1 displays live *E.coli* colonies in their biofilm.

Figure 4 shows CLSM images of *S.aureus* colonies present on samples SMP1–SMP4, organized as in Figure 3. We observed similar trends of bacterial colonization with respect to *E.coli* strain. Also in the case of *S.aureus* the increase of nanometer-scale roughness causes a significant reduction of the biofilm formation and in the number of attached bacteria.

**Figure 5:**
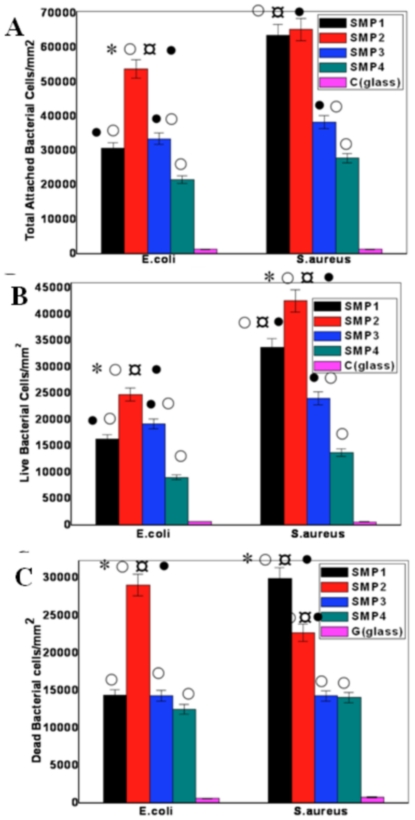
Quantification of attached bacterial colonies on ns-TiO_2_ samples with varying morphology. A) Total bacterial count of *E.coli* and *S.aureus* species showing significant higher adhesion on sample SMP1 and SMP2 compared to sample S3 and S4 and control glass coverslip. The two species show nearly similar trends for both live (B) and dead (C). Data shown are mean ± SEM. (* Compared to sample SMP1 (50 nm); ¤ Compared to sample SMP3 (200 nm); •compared to sample SMP4 (300 nm); ○compared to control (glass).

In order to study quantitatively the effect of surface morphology on bacteria adhesion we analyzed the data presented in Figures 3 and 4 quantifying the number of live/dead cells as a function of nanoscale roughness (Figure 5). The total number of cells attached onto any substrate (live+dead cells) were counted (Figure 5A). We found significantly higher total number of *E.coli* cells attached on sample SMP2 compared to any other ns-TiO_2_ film (t≤0.02 for all sample, p≤0.05). In case of *S.aureus* strain, almost equal number of cells colonize samples SMP1 and SMP2, and this number was significantly higher than on other samples, including control (t≤0.01 for all samples, p≤0.05).

Figure 5B displays live cells on ns-TiO_2_. A higher number of cells of both strains colonize on sample SMP2 (t = 0.03 and 0.01 for *E.coli* and *S.aureus* respectively, p≤0.05). Significantly less cells were present on sample SMP4 compared with all other nanostructured samples, other than control, where the number of attached cells is the least. In case of remnant dead cells on different substrates (Figure 5C), significantly higher number of *E.coli* colonies harbor on SMP2 (t≤0.001 for all sample, p≤0.05). Sample SMP2–SMP4 harbor nearly equal number of dead *E.coli* cells, while least dead cells recorded on control glass substrates. In the case of *S.aureus* strain, sample SMP1 is colonized by the highest number of dead cells (t = 0.01 compare to SMP2, and 0.001 compare to all other substrates, p≤0.05). Thus, on the basis of these results, we conclude that SMP2 sample supports maximum number of cell adhesion irrespective to the strain.

### Biofilm formation on nanostructured samples

In order to study how nanometer-scale morphology influences biofilm formation, we performed quantitative analysis of the structural parameters that characterize biofilms on the different nanostructured samples using Confocal Laser Scanning Microscopy (CLSM).

**Figure 6:**
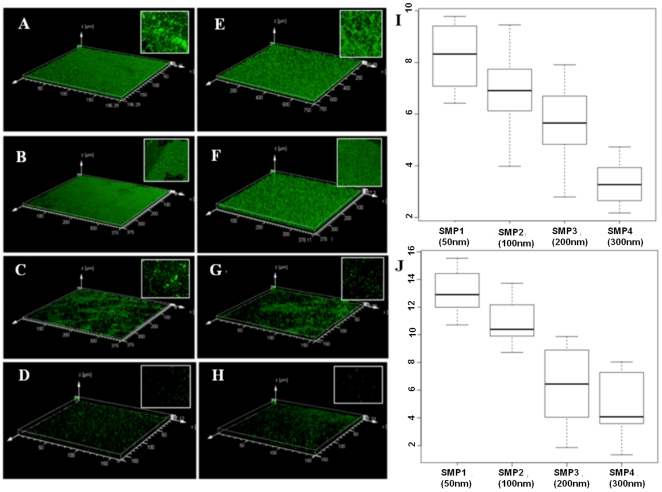
The CLSM biofilm architecture of *E.coli* (A–D; left column) and *S.aureus* species (E–H; right column). CLSM 3D topographic reconstruction showing bacterial microcolonies encapsulated in EPS forming thick biofilm on SMP1&SMP2 and scattered patches of microcolonies on SMP3&SMP4 (stained green with B-35000, BacLight green live bacterial stain). Inset in each image show 3D projections of biofilm structure obtained confocal z-stack using IMARIS 7.0, Bitplane's core software. Extreme right panel depicts Box and Whisker diagram of bacterial biovolume of *E.coli* (I) and *S.aureus* (J). A box represents 25^th^ to 75^th^ percentile range, intersected by median line. Whiskers extend above and below the box range, indicating highest to lowest values.

We found a marked variability in three dimensional biofilm architecture on different nanostructured titania films between the two species as shown in Figure 6A–D (*E.coli*) and Figure 6E–H (*S.aureus*). The samples with lower nanoroughness show a thick biofilms formation whereas sample SMP3 with larger, intermediate roughness, showed bacterial cells attached to surface in abundance, although no biofilm formation. Sample SMP4 with highest thickness and Rq, shows fewer cells attached.

Figure 6 shows distinctive biofilm features of *E.coli* and *S.aureus* respectively. Quantitative structural parameters of the biofilms, such as biovolume and thickness, were extracted from confocal stack images and analyzed as described elsewhere [35]–[36]. The measurement of biovolume and thickness of biofilm revealed that *S.aureus* species formed thick and rough biofilm, with higher biovolume compared with *E.coli* species. From three-dimensional topographic CLSM images one can note that both bacterial species show significantly thicker biofilm formation on thin ns-TiO_2_ samples with lower nanoscale surface roughness (SMP1 and SMP2), whereas biofilm formation is inhibited on thicker and rougher ns-TiO_2_ films (SMP3 and SMP4) (t≤0.03 for SMP1 and SMP2 versus SMP3 and SMP4). Figure 6C,D and Figure 6G,H (showing images acquired on samples SMP3–SMP4) clearly exhibit only a few, small scattered cell clusters with big voids without colonies, as opposite to Figures 6A,B and 6E,F (showing images acquired on samples SMP1–SMP2). Insets in each figure show 3D projections of biofilm architectures. From insets it is evident that thin ns-TiO_2_ samples form compact and carpet-like thick sheet of bacterial biofilm on the entire surface available, whereas thicker nanostructured samples show less prominent adhesion characteristics. The inhibitory role of roughness becomes more evident from scattered clusters of highly fluorescent cell aggregates, in between big gap and voids, which represent complete absence of bacterial microcolonies.

Figure 6I–J show a comparison between mean biovolumes of the biofilms of *S.aureus* and *E.coli* on different nanostructured films. *S.aureus* revealed maximum structural variability of biofilm characteristics; moreover, the mean biovolume for *S.aureus* was significantly higher compared with *E.coli* species (t<0.01 for both *sps.*, p≤0.05). Examining the effect of Rq and thickness on biovolumes, the mean biovolumes were noted to be significantly higher for thin ns-TiO_2_ films with lower R_q_ (t<0.01 for both *sps.*, p≤0.05).

**Figure 7:**
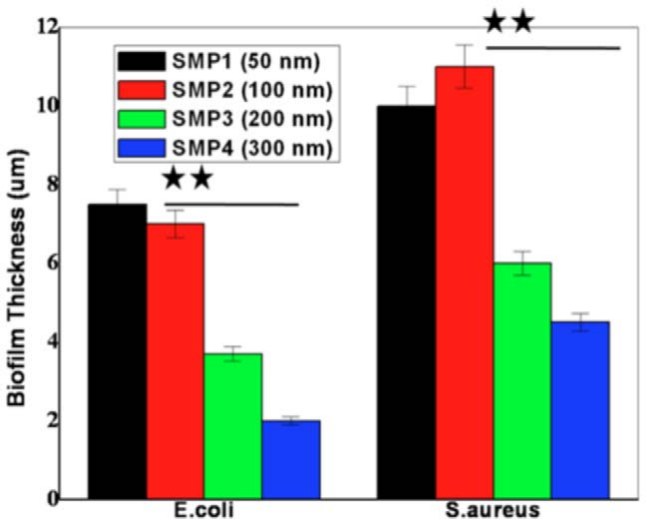
Quantification of biofilm thickness. Quantitative biofilm thickness of *E.coli and S.aureus* depicting significant thick biofilm formation on 50 nm and 100 nm ns-TiO_2_ samples compared to 200 nm and 300 nm samples. Data represents mean ± SEM; n = 3.

Figure 7 reports biofilm thickness values for the two species. There is a significant difference in biofilm thickness between SMP1/ SMP2 to SMP3/SMP4 samples (t<0.02 for both *sps.*, p≤0.05). The thickness of biofilm structure mainly accounts the amount of the EPS produced that harbors the microcolonies of bacteria. Thus, quantification reveals that lower surface roughness induces more EPS productions, henceforth thicker biofilm structure which is in line with previous reports [37].

## Discussion

In order to quantitatively assess how nanoscale morphology influences bacterial adhesion and biofilm formation, we produced nanostructured titania films with controlled surface morphology. The main advantage of the SCBD method compared with other methods for the production of nanostructured surfaces, such as chemical etching or mechanical roughening, is that film morphology can be controlled reliably by controlling the thickness of the deposited films, without affecting surface chemical composition [24]–[26]. An important factor influencing bacteria adhesion is protein adsorption: when biomaterial surfaces interact with biological systems *in vitro* or *in vivo*, they are immediately coated by the proteins present in the biological media [11]–[13]. This adsorbed protein layer mediates the interaction between the surface and cells, translating surface chemical and physical properties into a biological language. Our data show that the increase of nanoscale roughness (from 16 nm to 32 nm) causes a significant increase of FBS protein adsorption (Figure 2A). In particular, rougher samples SMP3 and SMP4 show a significant higher amount of adsorbed proteins than samples SMP1 and SMP2. As we already demonstrated in previous studies, the increase of the amount of adsorbed proteins on cluster-assembled titania is tailored by surface morphology that promotes protein nucleation on the surface in correspondence of nanometric surface pores with aspect ratio higher than a specific threshold value [15]. In cluster-assembled titania when roughness increases the numbers of pores, where nucleation occurs, also increases, thus causing a significant increase of proteins adsorption. The characterization of nanostructured samples demonstrated that both SE and WCA are influenced by nanoscale morphology; in fact, when roughness increases we measured an increase of SE and a decrease of WCA (i.e. increase of surface hydrophilicity) (table 1). Similar effects have been previously reported by our group [24]. SE and WCA are also influenced by surface morphology. The increase of surface wettability may have also an influence on the amount of adsorbed proteins, since a very hydrophobic surface may prevent water from wetting extensively the available surface, keeping protein away from it. On the other hand, the increase of surface hydrophilicity may reduce the hydrophobic interaction between proteins and the surface, causing a lower adsorption affinity. These observations and also from our previous studies [15], let us think that the (modest) increase in wettability induced by the increase in surface roughness is not the driving parameter of the protein adhesion enhancement. In turn, the main factor that determines the measured protein adsorption profile is nanometer scale morphology through the aforementioned nucleation effect.

We observe that bacteria adhesion on nanostructured titania is strongly dependent on surface morphology and on the amount of adsorbed proteins. The number of adhered bacteria as a function of surface roughness follows a trend that anti-correlates with the amount of adsorbed proteins on the nanostructured samples (Figure 5). In fact, bacteria cells showed preferential attachment on less rough samples SMP1 and SMP2, while rougher samples SMP3 and SMP4, where maximum protein adsorption was noted, showed comparatively less bacterial cells attached and no biofilm formation. Two concomitant effects can explain these results. When roughness increases the formation of proteins clusters on the surface creates a thick protein layer that may significantly suppress bacteria adhesion. In fact, adsorbed proteins may act as a passivation layer, which inhibits bacteria adhesion, as observed in previous studies on flat biomaterial surfaces [20], [38]–[39]. Moreover, we demonstrated that cluster-assembled titania surface morphology is significantly changed by the adsorption of proteins (Figure 2B,C). AFM images show that after protein adsorption, ns-TiO_2_ surface gets significantly smoother due to the partial filling of surface pores, which determines a significant reduction of R_q_ from 25 to 17 nm. Therefore, the nucleation of proteins that are present in the bacteria culture medium may reduce significantly the morphological difference between SMP1/SMP2 and SMP3/SMP4 (where nucleation is more pronounced). As a consequence, any possible (positive) influence of nanometer scale morphology on bacterial adhesion is also significantly suppressed.

These two effects, related to nanoscale morphology, cause a reduction of bacteria adhesion when roughness is increased from 21 nm (SMP2) to 25 nm (SMP3). Interestingly, these inhibitory effects have never been reported in previous studies, where some authors have instead demonstrated that nanometer scale morphology promotes bacterial adhesion and proliferation [17]–[20]. These contrasting results can be explained if we consider that:

- the decrease of bacterial adhesion, as discussed above, is related to the increase of the amount of adsorbed proteins, which passivate the surface and inhibit cell attachment;
- the mechanism of protein adhesion enhancement is more complex than a simple geometrical amplification due to the increased available surface because of increasing corrugation. Rms roughness, which is the standard parameters used for characterizing nanostructured surfaces, is not the driving parameter.

Therefore inhibition of bacteria adhesion may not be observed in surfaces having the same roughness of those used in this study, because other less apparent morphological parameters are different. In particular, the surface features directly influencing protein adsorption are pores with nanometric dimensions and suitable aspect-ratio that promote protein nucleation, significantly increasing the surface protein loading [15]. These structures are highly abundant on cluster-assembled titania surfaces with higher roughness, but are absent on surfaces produced with different methods, such as chemical etching or mechanical roughening. When in turn morphology does not promote protein accumulation onto the surface, the increase of surface roughness effectively promotes bacterial adhesion, as described in previous studies. In fact, this is what we observe on samples SMP1 and SMP2. These samples adsorb the same amount of proteins but have different roughness; sample SMP2, with higher roughness, promotes higher cell attachments and biofilm formation compared to the smoother sample SMP1. Nanoscale grain boundaries may be responsible for guiding higher bacterial adhesion on samples [40]–[41].

Our experiments indicate that also biofilm formation is dependent on morphology and on protein adsorption. In fact, when roughness increases, biofilm thickness and volume significantly decrease. Remarkably, the two bacterial species show similar propensity to adhere onto nanostructured substrates in spite of the differences in their size, shape and cell wall characteristics (*E.coli*: gram negative, rod shaped; *S.aureus*: gram positive, cocci), and to the fact that *S.aureus* shows more EPS production of biofilm than *E.coli* ( in agreement with similar observation of EPS production by other gram positive species on comparatively smoother surfaces [37].

As a further confirmation of the key role of surface morphology on bacterial adhesion, we observe that since measured zeta potential of both species were similar (*E.coli*: 40.2±3 mV; *S.aureus*: 34.8±3 mV), and being likely similar the surface chemistry of all ns-TiO_2_ samples (as revealed by XPS), we exclude important electrostatic contributions to bacteria adhesion.

### Conclusions

We characterized the interaction of *E.coli* and *S.aureus* with cluster-assembled titania surfaces demonstrating a precise and quantitative relationship between surface nano-morphology and bacterial adhesion.

Our data show that bacterial adhesion and biofilm formation depend on nanoroughness in a non-monotonous way. After a first linear increase of bacterial adhesion with surface roughness at low corrugations, we observed a significant decrease of bacterial biofilm formation and adhesion with the further increase of roughness. Interestingly, the number of adhered bacteria anti-correlates with the measured amount of adsorbed proteins on the nanostructured samples. In fact, the accumulation of proteins on the rougher surfaces downplays bacterial adhesion and biofilm formation by creating a thick layer, which reduces the interaction of bacateria with the nanostructured surface, inhibiting bacteria adhesion (passivation effect). Moreover, the protein layer significantly flattens the surface, suppressing any possible effect of the nanoscale morphology on bacteria adhesion (flattening effect).

The morphological parameters that drive the increase of the amount of adsorbed proteins when roughness increases, and the consequent reduction of bacteria adhesion, are the dimension and aspect ratio of the surface nanometric pores. Our research demonstrates that roughness (specific area) is not the only morphological parameter that affects and controls bacteria adhesion. This finding indicates that in perspective it could be possible to tailor surface morphology of titanium biomedical implants to promote mammalian cell interaction while inhibiting bacterial colonization.

## Materials and Methods

### Nanostructured TiO_2_ thin film synthesis and characterization by Atomic Force Microscopy

Nanostructured TiO_2_ films were deposited by a supersonic cluster beam deposition (SCBD) apparatus equipped with a pulsed microplasma cluster source (PMCS) [21]–[23]. The PMCS operation principle is based on the ablation of a titanium rod by an argon plasma jet, ignited by a pulsed electric discharge. After the ablation, Ti atoms and ions thermalize with argon and condense to form partially oxidized clusters. The mixture of clusters and inert gas is then extracted in vacuum through a nozzle to form a seeded supersonic beam, which is collected on a set of standard glass microscope slides located in the beam trajectory. The clusters kinetic energy is low enough to avoid fragmentation and hence a nanostructured film is grown. Rms-roughness of nanosctructured titania films can be typically controlled during deposition in the range 5–40 nm, with corresponding specific areas (the ratio of the surface to the projected area) in the range 1–2. Film thickness is typically in the range 10–400 nm. We deposited four different samples of ns-TiO_2_ thin films with different thickness: 50 nm, 100 nm, 200 nm and 300 nm (SMP1–SMP4). All deposition were made on round glass coverslips (13 mm diameter, 0.13–0.16 mm thickness, Electron Microscopy Sciences) using stencil masks placed in front of the substrate. The post-deposition thermal treatments have been carried out in a muffle furnace at 250°C for 2 hours in ambient air atmosphere.

Surface morphology of the cluster-assembled ns-TiO_2_ thin films were characterized in air by atomic force microscopy (AFM) using a Nanoscope multimode IV (Veeco Instruments) in tapping mode with a single-crystal silicon tip with nominal radius of curvature 5–10 nm and cantilever resonance frequency ∼300 kHz. Scan areas were 2×1 µm^2^ (2048×1024 points) with scan rates of 1–2 Hz. Film thickness was calculated by surface profilometry (P-16^+^™, KLA-Tencor; San Jose; CA;USA) across a sharp step produced by masking the film during the deposition, and cross checked by AFM. For profilometric calculations, three samples of each surface type were briefly scanned to evaluate the overall homogeneity of the surface at five different locations. As shown in Table S1, thickness values measured by AFM and stylus profilometer agree within the experimental error. AFM values deviate by the nominal values by a few % only, therefore in the manuscript the nominal values are reported. The average nanoscale root-mean-square (rms) roughness (Rq), Skewness R_sk_, Kurtosis R_ku_, and specific area (A_spec_) parameters were calculated from AFM images using MATLAB routines according to the definitions and formulae reported in Text S1. Morphological parameters were calculated with 0.8 mm Gaussian filter cutoff.

### Surface energy and contact angle measurements

We used the sessile drop method to measure the static contact angles of on ns-TiO_2_ surfaces. Contact angle measurements were performed using an FTA1000 (First Ten Ångstroms Inc.) instrument. For statistical validation of results, each measurement of a particular contact angle was recorded in 150 images taken within 5 s with a Pelco Model PCHM 575-4 camera (standard deviation ∼2°, unless otherwise stated); images analysis was performed by the FTA Windows Mode 4 software. Details on the calculation of surface energy (SE) from contact-angle data are provided in Text S1.

### X-ray photoemission spectroscopy (XPS)

The ns-TiO_2_ was characterized in a UHV apparatus Leybold LHS 10/12 equipped with a hemispherical electron analyzer and conventional X-ray source (Al Kα = 1486.7 eV). The high resolution spectra were acquired in constant pass-energy mode E_pass_ = 30 eV. The overall energy resolution was 1.0 eV. The pressure in the experimental chamber during experiments was below 1·10^−9^ mbar. All spectra are referenced to the Fermi level and the binding energy scale is calibrated via the Au 4f_5/2_ core level line (located at 88.5 eV) of a clean polycrystalline Au sample. No charging effects on the samples under investigation were observed during all the measurements. The line shapes were fitted with mixed singlets obtained by a linear combination of a Gaussian and a Lorentzian profiles sited on a Shirley background.

### Protein adsorption experiments

One ml droplet of the Dulbecco's minimum essential medium (DMEM, Gibco) containing 10% fetal bovine serum (FBS, Invitrogen) was deposited on ns-TiO_2_ coated glass cover slip in a 12 well cell culture plate. After incubation for 30 minutes at 37°C, the samples were transferred to a new 12-well plate (one ns-TiO_2_ coated glass cover slip/well) and washed thrice with 1 ml PBS. 500 ml of 1% sodium dodecyl sulfate (SDS) solution were added to these wells and shaken for 1 h to detach proteins from the disk surfaces. The protein concentrations in the collected SDS solutions were determined using a MicroBCA protein assay kit (Pierce). The optical density of the samples was measured spectrophotometrically at 562 nm against a standard protein calibration curve as per the manufacturer's protocol and surface density of protein converted to per square unit area. Three independent adsorption studies were performed in triplicate over all test and reference samples.

BSA lyophilized powder (Sigma Aldrich) was dissolved in PBS at 27.5 uM concentration. Ns-TiO_2_ samples were typically incubated for 1 h with 400 mL of BSA solution. For AFM imaging (tapping mode in air, as described before), samples were washed 3 times for 1 minute in PBS and 3 times in bidistilled H_2_O for 1 minute, dried using a gentle nitrogen flux, and imaged before and after proteins adsorption.

### Bacterial culture and Adhesion profile

Gram positive *E.coli* and gram negative *S.aureus* was obtained from National Collection of Industrial Microorganism (NCIM), a microbial cell repository of National Chemical Laboratory (NCL), Pune. *E. coli* and *S.aureus* were inoculated from bacterial stocks obtained and grown at 37°C in standard Luria broth (LB) under aerobic condition for 24 hrs in an orbital shaker, until optical absorbance reached the value OD_600_ = 0.8. The cell density of each species was adjusted at OD_600_ = 0.2, to ensure that the all the ns-TiO_2_ samples had similar numbers of cells without variations in the cell densities [42]. For the bacterial adhesion and biofilms formation studies, prior to seeding, sterilized 13 mm diameter glass coverslips coated with and without ns-TiO_2_ film were placed into a standard 12-well culture plate and were washed twice with phosphate buffer saline (PBS). Sterile ns-TiO_2_ coverslips in culture plate were inoculated with 20 µl fresh culture diluted at a ratio of 1∶90 in Dulbecco's Modified Eagles Medium (DMEM, Hyclone; Logan, UT/USA) and left at 30°C for 24 hrs with constant shaking at 200 rpm to prevent settling of the cell solution. DMEM were supplemented with 10% fetal bovine serum (FBS, Hyclone), 1% penicillin–streptomycin (P/S, Hyclone), 50 mg/ml L-ascorbate acid (Sigma Aldrich), and 10 mM b-glycerophosphate (Sigma Aldrich). Four samples of ns-TiO_2_ films and one clean glass coverslip were used for each species. Bacterial cells were collected at the logarithmic stage of growth. After incubation, the bacterial coated ns-TiO_2_ samples were gently washed with MilliQ water to remove non-adherent cells and left to dry at room temperature for 30 min at 50% humidity. Bacterial surface charge was measured by electrophoretic mobility test which provides the zeta potential of the surface and assumed it to be equivalent to bacterial surface charge [43]. Bacterial cells cultured as described above were resuspended in 1 mM PBS solutions, and zeta potentials measured using 20 cycles per analysis (ZetaPALS analyzer, Brookhaven Instruments Corp). Three independent experiments performed in triplicate (n = 3) were conducted to confirm the results. The samples for microscopic imaging were prepared by standard procedures with optimum care to avoid any modification to the distribution and the orientation of bacteria over ns-TiO_2_ surface, influencing cell parameter quantification or cell retention on the surface. This is important because bacterial imaging results are affected with the hydrodynamics conditions and the methods of fixation and drying of the cells for CLSM and AFM images has been confirmed prior to sampling [44].

### Quantification of bacterial density over nanostructured surface

Bacterial density (total bacteria colonies) over the nanostructured surfaces were determined by summing the number of live and dead bacteria colonies quantified using ImageJ. In order to image the viable bacterial count and the extracellular polymeric substance (EPS), established microbial staining techniques were adopted. For live/dead bacterial count, after 24 hour incubation of ns-TiO_2_ sample-bacteria on shaker in DMEM media as described above, the substrates were rinsed twice with Tris-buffered saline (TBS) comprised of 42 mM Tris–HCl, 8 mM Tris Base, and 0.15 M NaCl (Sigma Aldrich). Then incubated for 15 min in dark with the BacLight Live/Dead solution (Molecular Probes Inc., Leiden, The Netherlands) dissolved in TBS at the concentration recommended by the manufacturer, 50% glycerol solution in TBS, visualized and counted in situ using Confocal Laser Scanning Microscopy (CLSM) microscope (LEICA TCSSP2AOBS) with a water immersion objective lens at 40× magnification, zoom 1∶5 and image analysis were performed with ImageJ NIH image processing software [45].

In brief, the imageJ tool enabled us to quantify the bac-light stained live-dead bacterial cells attached on ns-TiO_2_ using four basic steps. We made background corrections and change the scale of the image to micrometer to spatially calibrate the image using line selection tool (Analyze>Set Scale). Next, we converted the image into grey scale by using Image>Type>RGB Stack command which split the image in the 3 channels (no blue channel in our case). Subsequently, relative proportion of the live bacterial cells (green) against the dead (red) microbiota was estimated by counting fluorescence specific pixels in digital fluorescent images using the ImageJ software. This software classifies and counts particles on the basis of their relative density (fluorescence) compared with the background via a threshold process. As few bacteria were in aggregates, counting of individual cells was not possible. To overcome this, we adopted Auto threshold function to analyze particles, filtering out those particles that are too big (large clumps) or too small. Subsequently, we segmented (isolate) the red-green stained dead-live bacterial cells after thresholding using the montage of the red-green channel to quantify the stained bacterial cells by measuring the threshold area and area fraction tools (*Analyse>Measure*). The analysis was performed on fives images on each sample from different locations of the bacterial colonies comprising 3 independent experiments. Averages and standard deviations of proportions were calculated.

### Biofilm formation and nucleic acid labeling with fluorescent marker for quantification

A subculture was carried out for overnight, subsequently a 250 µL volume of this subculture adjusted to an OD_600_ nm = 0.2, was added to the 12 well culture plate containing glass cover slips coated with different thickness/Ra of ns-TiO_2_. After 24 hrs of adhesion at room temperature (RT), the substrate was rinsed with 150 mM NaCl in order to eliminate any non-adherent bacteria before and 250 µL TSB was further added to the culture. All of the different substrates were incubated for 24 h at RT. After the development of biofilms, the substrates were rinsed with 150 mM NaCl and 5 µM of Syto9 (GFP; absorption:488 nm, emission: 500–600 nm range) were added to TSB containing a cell permeable green fluorescent nucleic acid marker (1∶1000 dilution of Syto9 in 5 mM in DMSO; Invitrogen, CA). The culture plate with ns-TiO_2_ samples was then incubated in the dark at RT for 30 minute to enable the fluorescent labeling of the attached bacterial cells. Samples were visualized and biofilm structural properties were quantified in situ using confocal laser scanning (CLSM) microscope (LEICA TCSSP2AOBS) at 40× magnification of water immersion objective lens with a 0.8 N.A., zoom 1∶5. The average z-stacks of 1 µm were acquired from each biofilm horizontal plane with maximum of five stacks at different field of view. Three-dimensional projections of biofilms structure were reconstructed using the Easy 3Dfunction of the IMARIS 7.0, Bitplane's core software (Saint Paul, Minnesota, USA). The quantification of biovolume of encapsulated bacterial cells in EPS matrix, representing overall volume of cells in biofilm (µm^3^), was carried out using free PHLIP [34].

### Statistical analysis

For the quantification, data was represented by the mean value with the standard error of the mean (SEM) as per commonly used protocols. Statistical analysis was conducted using a paired t-test or an unpaired, two-tailed Student's t-test. P-value of <0.05 was considered to be statistically significant. For the statistical validation of data, three independent experiments (n = 3) were performed in triplicate.

## Supporting Information

Figure S1 **XPS analysis of ns-TiO_2_ films.** XPS spectra of annealed ns-TiO_2_ film: a) O 1s and b) Ti 2p edge. The samples appear to be fully oxidized as titanium dioxide.
(PDF)

Figure S2 **Water Contact Angle (WCA) measurements on ns-TiO_2_ films.** Photographs of water droplets sitting on the surfaces of ns-TiO2 films with different surface morphology. A: SMP1; B: SMP2: C: SMP3; D: SMP4.
(PDF)

Figure S3 **WCA and AFM analysis of reference glass substrate.** Surface characteristics of reference substrate glass. A: WCA profile; B: AFM characteristics (for quantitative parameters, see text).
(PDF)

Table S1 **Thickness of ns-TiO_2_ films measured by AFM and stylus profilometer.** The thickness values measured by AFM and stylus profilometer [1] agree within the experimental error and are very close to the nominal values (h = 50, 100, 200, 300 nm).
(PDF)

Text S1 **Supplementary Methods.** Calculations of morphological parameters from AFM images. Contact angle measurements and surface energy calculation on ns-TiO_2_ films. Bacterial live-dead cell counting using ImageJ.
(PDF)

Movie S1 **Dynamic motility of** ***E. coli*** **biofilm.** This movie shows the dynamic motility of *E. coli* biofilm “spread sheet” grown in vitro on 50 nm thick ns-TiO_2_ films. The sample was incubated for 3 days and examined in situ in real time by confocal microscopy. The movie shows sections from top to bottom through the sheet. In the movie the motile clusters of cells into the surrounding medium can be seen. Viable *E.coli* cells are stained red with SYTO® 17 Red.
(AVI)
