# Supplementary figures and images for: Quantitative Characterization of the Influence of the Nanoscale Morphology of Nanostructured Surfaces on Bacterial Adhesion and Biofilm Formation
Source: PLoS One. 2011 Sep 26;6(9):e25029. doi: 10.1371/journal.pone.0025029 (PMC3180288; doi:10.1371/journal.pone.0025029)

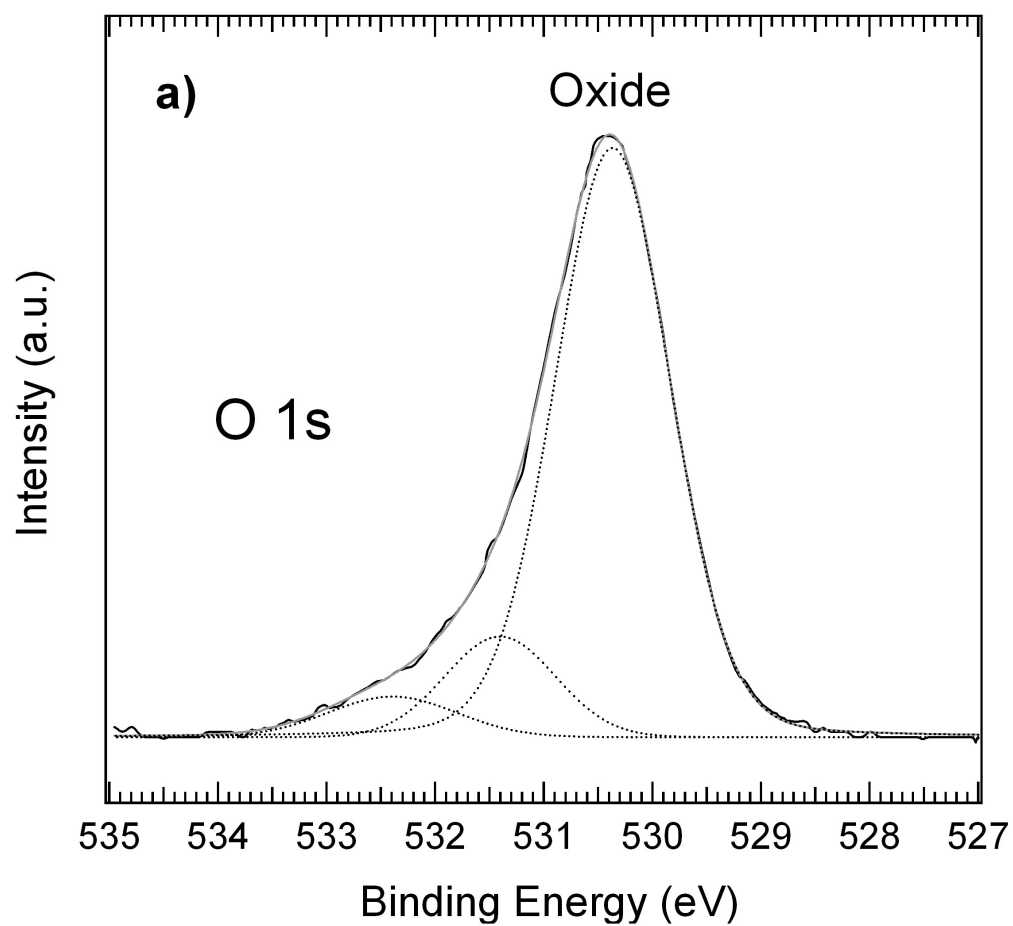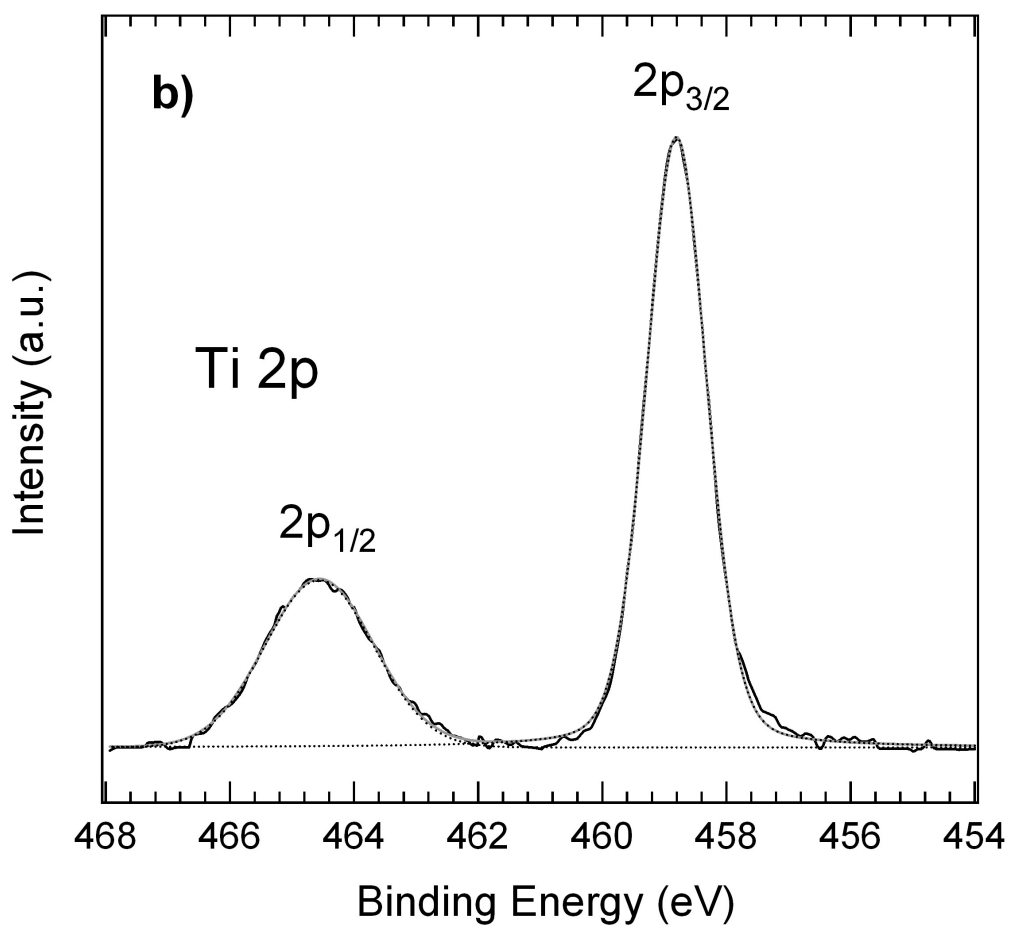

Supplement: Figure S1 — XPS analysis of ns-TiO2 films. XPS spectra of annealed ns-TiO2 film: a) O 1s and b) Ti 2p edge. The samples appear to be fully oxidized as titanium dioxide. (PDF) [file pone.0025029.s001.pdf]

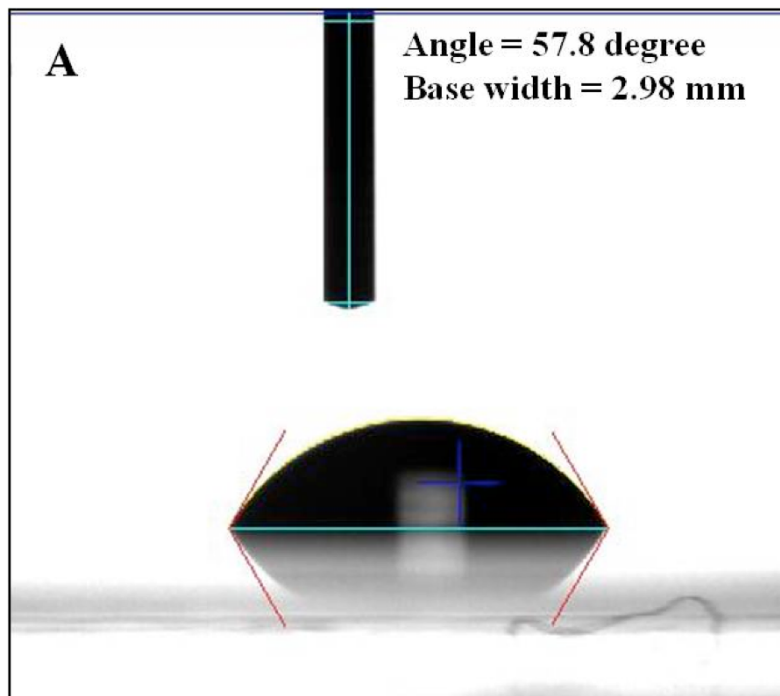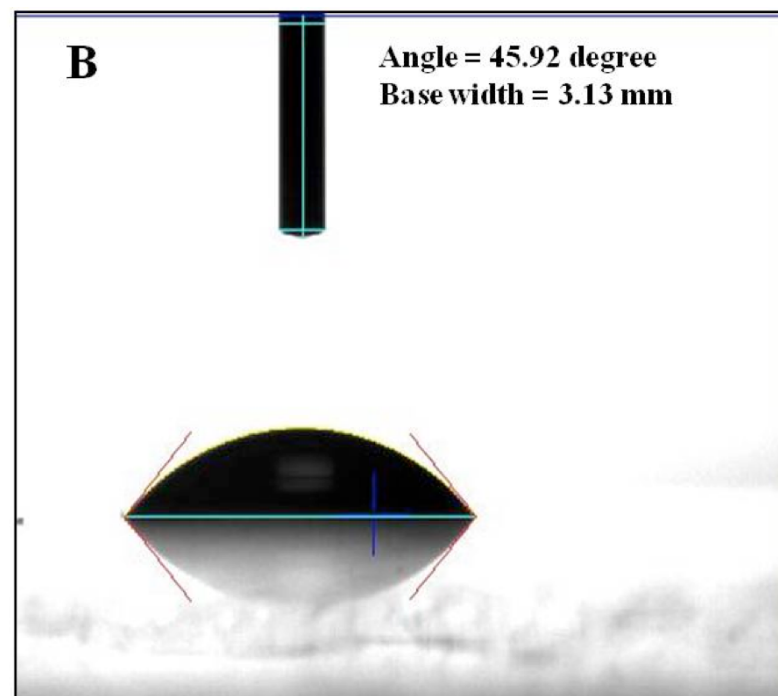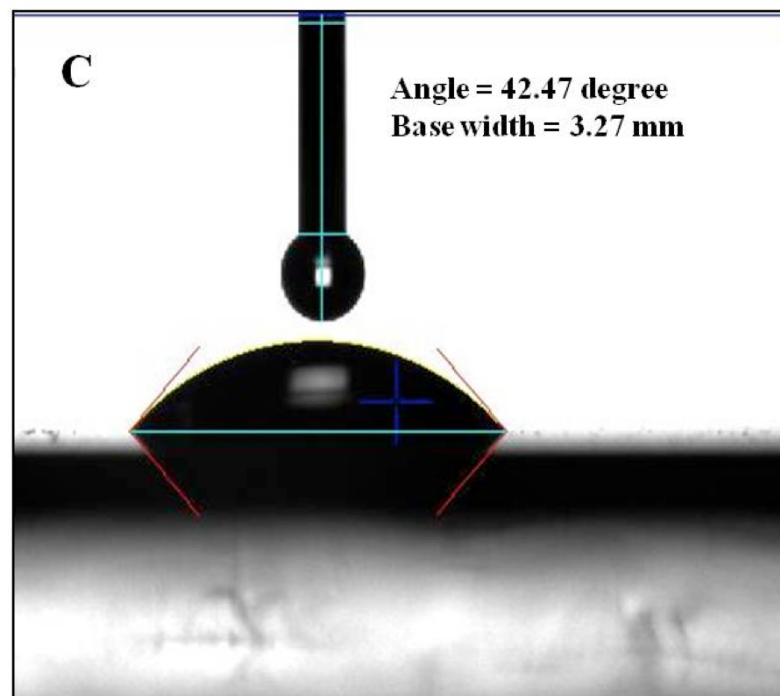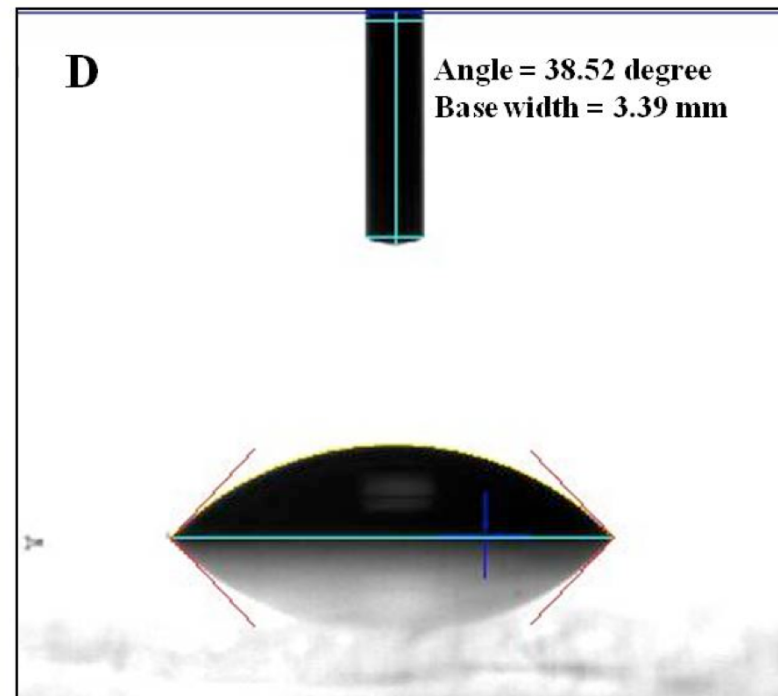

Supplement: Figure S2 — Water Contact Angle (WCA) measurements on ns-TiO2 films. Photographs of water droplets sitting on the surfaces of ns-TiO2 films with different surface morphology. A: SMP1; B: SMP2: C: SMP3; D: SMP4. (PDF) [file pone.0025029.s002.pdf]

Angle = 20,32 degrees  
Base Width = 4,4746mm

**A**

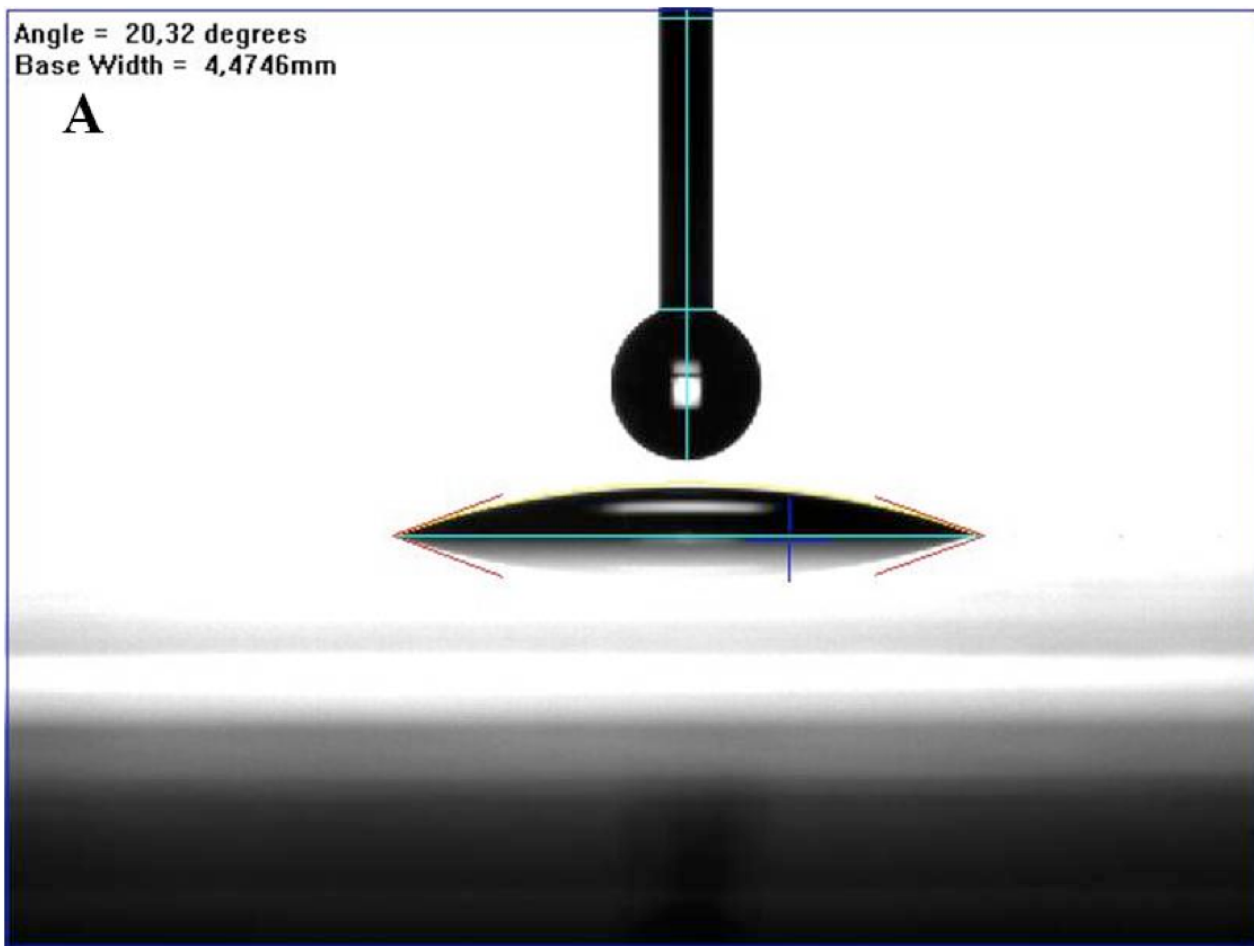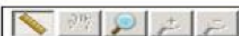

**B**

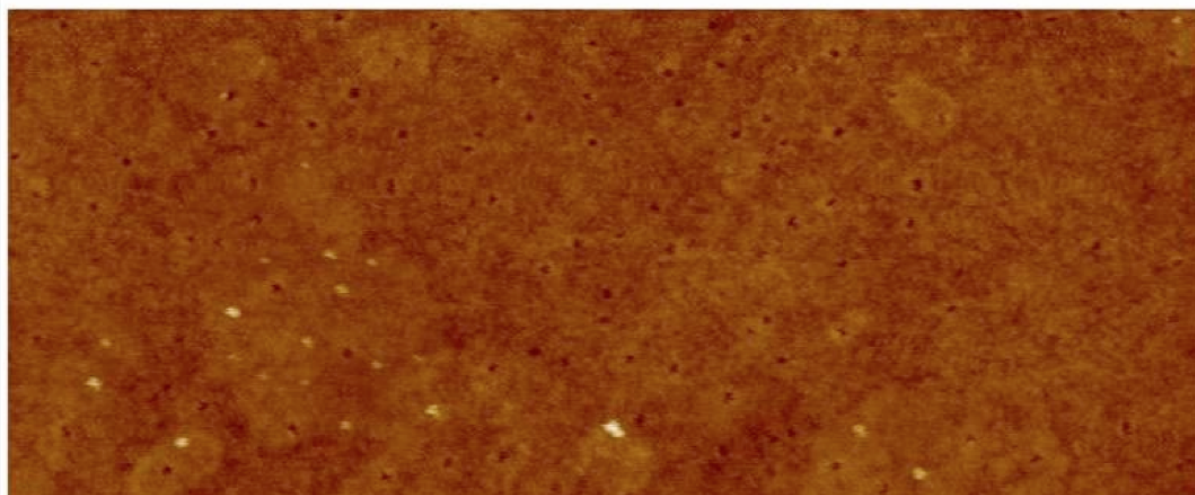

0.0

Height

2.0  $\mu\text{m}$

Supplement: Figure S3 — WCA and AFM analysis of reference glass substrate. Surface characteristics of reference substrate glass. A: WCA profile; B: AFM characteristics (for quantitative parameters, see text). (PDF) [file pone.0025029.s003.pdf]
