# Supplementary material for: Quantitative Characterization of the Influence of the Nanoscale Morphology of Nanostructured Surfaces on Bacterial Adhesion and Biofilm Formation
Source: PLoS One. 2011 Sep 26;6(9):e25029. doi: 10.1371/journal.pone.0025029 (PMC3180288; doi:10.1371/journal.pone.0025029)
Supplement: Table S1 — Thickness of ns-TiO2 films measured by AFM and stylus profilometer. The thickness values measured by AFM and stylus profilometer [1] agree within the experimental error and are very close to the nominal values (h = 50, 100, 200, 300 nm). (PDF) [file pone.0025029.s004.pdf]

Table S1. Thickness of ns-TiO_2 films measured by AFM and stylus profilometer

The thickness values measured by AFM and stylus profilometer [1] agree within the experimental error and are very close to the nominal values (h = 50, 100, 200, 300 nm).

Sample	Nominal thickness (nm)	Thickness by Profilometer (nm)	Thickness by AFM (nm)
SMP1	50	64.1 ± 6.4	54 ± 6
SMP2	100	115.9 ± 8.8	105.2 ± 3.9
SMP3	200	197 ± 6.8	202 ± 5.1
SMP4	300	309 ± 7.3	296.7 ± 5.5
