# Supplementary material for: Quantitative Characterization of the Influence of the Nanoscale Morphology of Nanostructured Surfaces on Bacterial Adhesion and Biofilm Formation
Source: PLoS One. 2011 Sep 26;6(9):e25029. doi: 10.1371/journal.pone.0025029 (PMC3180288; doi:10.1371/journal.pone.0025029)
Supplement: Text S1 — Supplementary Methods. Calculations of morphological parameters from AFM images. Contact angle measurements and surface energy calculation on ns-TiO2 films. Bacterial live-dead cell counting using ImageJ. (PDF) [file pone.0025029.s005.pdf]

Quantitative characterization of the influence of nanoscale morphology of nanostructured surfaces on bacterial adhesion and biofilm formation

Ajay Vikram Singh[1,2], Varun Vyas[1,2], Rajendra Patil[3], Vimal Sharma[1,4], Pasquale Emanuele Scopelliti[2,4], Gero Bongiorno[4], Alessandro Podestà[2] , Cristina Lenardi[2,4], *Wasudev Namdev Gade[3], *Paolo Milani[2,4].

[1] European School of Molecular Medicine (SEMM), IFOM-IEO Campus, via Adamello 16, 20139, Milano, Italy.

[2] Interdisciplinary Centre for Nanostructured Materials and Interfaces (CIMAINA) and Department of Physics, via Celoria 16, Università degli Studi di Milano, 20133, Milano, Italy.

[3] Department of Biotechnology, University of Pune, Ganesh Khind, Pune-411007, India.

[4] Micro and Nano Fabrication Platform, Fondazione Filarete, Viale Ortles 22/4, 20139 Milano, Italy.

* Corresponding authors. E-mail: pmilani@mi.infn.it; wngade@unipune.ernet.in

Supporting Text S1

Table of Contents

1 - Calculations of morphological parameters from AFM images

2 - Contact angle measurements and surface energy calculation on ns-TiO2 films

3 - Bacterial live-dead cell counting using ImageJ

References

1 - Calculations of morphological parameters from AFM images

Several statistical morphological parameters have been calculated from AFM topographies: root-mean-square roughness (Rq), Skewness (Rsk), and Kurtosis (Rku), specific area A_{spec} [2]. Rq represents the rms deviation of height values from the mid plane value $\bar{h}$. For a Gaussian surface, about 68% of surface points have height between $\bar{h}$-Rq and $\bar{h}$+Rq. Gaussian surface possess a symmetric height values distribution, peaks and valleys being equally represented. Skewness (Rsk) is a statistical measure of the symmetry of the height distribution and it can highlight a prominence of valleys over peaks (Rsk<0), or vice versa (Rsk>0; for a symmetric surface, Rsk=0). Kurtosis quantifies the ‘peakedness’ of a surface. A surface with a Gaussian height distribution has a kurtosis value of 3; a surface with a narrower height distribution (sharp peaks and valleys over a rather flat background) has a kurtosis value greater than 3, while a surface that has a broader height distribution (rather continuous rounded asperities and valleys) has a kurtosis value of less than 3. AFM topographies are 2-dimensional arrays of height values, organized in rows and columns. Specific area is defined as the ratio of surface area to projected area; it is always larger than unity for rough surfaces. Statistical morphological parameters are calculated from AFM maps as listed below:

$$R_q = \sqrt{\frac{1}{N}\sum_{i,j}(h_{ij} - \bar{h})^2} \;;$$

$$R_{sk} = \frac{1}{NR_q^3}\sum_{i,j}(h_{ij} - \bar{h})^3 \;;$$

$$R_{ku} = \frac{1}{NR_q^4}\sum_{i,j}(h_{ij} - \bar{h})^4 \;;$$

$$A_{spec} = \frac{1}{N}\sum_{i,j}\sqrt{1+\nabla_{ij}^2} \,.$$

where h_{ij} represent the height values in the AFM topographic map (*i,j* are the row and column indices), *N* the total number of heights values in the map, $\bar{h} = \frac{1}{N}\sum_{i,j} h_{ij}$ if the average height of the

surface (the quota of the surface mid plane), and ∇_{ij}^{2} is the squared i,j-th element of the (discrete) surface gradient.

2 - Contact angle measurements and surface energy calculation for ns-TiO2 films

The Sessile Drop Technique was employed to measure contact angles of water and other liquids on ns-TiO2 films and to calculate the solid surface energies based upon the Owens/Wendt theory [3], which relates the dispersive and polar components of the surface energy (γ^d and γ^p) of the solid surface and the wetting liquid to the contact angle θ and the total surface energy of the liquid:

$$\gamma_L(1+\cos\theta) = 2\left(\sqrt{\gamma_L^d \gamma_S^d} + \sqrt{\gamma_L^p \gamma_S^p}\right)$$

The Owens-Wendt equation strictly holds for smooth surfaces; when applied to rough surfaces, the obtained surface energies should be considered as semi-empirical, not necessarily coincident with the thermodynamically-defined surface energies. In the case of rough ns-TiO2 surfaces, reported SE represent the effective surface energies, which characterize this material with respect to its chemical nature and specific surface topography. In Table 1 of the present work total surface energies are reported, as calculated by instrument software by summing different surface free energy contributions (Lifshitz–van der Waals, acid/base, electron acceptor and electron donor.

3 - Bacterial live-dead cell counting using imageJ

The imageJ tool enabled us to quantify the bac-light stained live-dead bacterial cells attached on ns-TiO_2 using four basic steps. We change the scale of the image to micrometer to spatially calibrate the image using line selection tool (Analyze>Set Scale). Next, we converted the image into grey scale by using Image>Type>RGB Stack command which split the image in the 3 channels (no blue channel in our case). Subsequently, we segmented (isolate) the red-green stained dead-live bacterial cells using thresholding and made the montage of the red-green

channel to quantify the stained bacterial cells by measuring the threshold area and area fraction tools (*Analyse>Measure*).

References

[1] Teague, E. C., Scire, F. E., Baker, S. M. & Jensen, S. W. Three-dimensional stylus profilometry. *Wear* 83, 1-12 (1982).

[2] Gadelmawla, E. S., Koura, M. M., Maksoud, T. M. A., Elewa, I. M. & Soliman, H. H. Roughness parameters. *Journal of Materials Processing Technology* 123, 133-145 (2002).

[3] D.K. Owens and R.C. Wendt, *J Appl Polym Sci* **13** (1969), p. 1741.
